# Prevalence and factors associated with severe undernutrition among under-5 children in Bangladesh, Pakistan, and Nepal: a comparative study using multilevel analysis

**DOI:** 10.1038/s41598-023-36048-w

**Published:** 2023-06-22

**Authors:** Mohammad Rocky Khan Chowdhury, Md Shafiur Rahman, Baki Billah, Mamunur Rashid, Melody Almroth, Manzur Kader

**Affiliations:** 1 Department of Epidemiology and Preventive Medicine, School of Public Health and Preventive Medicine, Faculty of Medicine, Nursing and Health Sciences, Monash University, Melbourne, Australia; 2 Research Center for Child Mental Development, Hamamatsu University School of Medicine, Hamamatsu, Japan; 3 United Graduate School of Child Development, Osaka University, Kanazawa University, Hamamatsu University School of Medicine, Chiba University, University of Fukui, Osaka, Japan; 4 Department of Public Health and Sports Science, Faculty of Occupational and Health Sciences, University of Gävle, Gävle, Sweden; 5 Institute of Environmental Medicine, Karolinska Institutet, Stockholm, Sweden; 6 Department of Medicine Solna, Clinical Epidemiology Division, Karolinska Institutet, Maria Aspmans Gata 30A, 17176 Stockholm, Sweden

**Keywords:** Epidemiology, Quality of life, Nutrition

## Abstract

Despite economic growth and poverty reduction, under-5 child undernutrition is still rampant in South Asian countries. This study explored the prevalence and risk factors of severe undernutrition among under-5 children in Bangladesh, Pakistan, and Nepal for comparison using the Composite Index of Severe Anthropometric Failure. We utilised information on under-5 children from recent Demographic Health Surveys. We used multilevel logistic regression models for data analysis. The prevalence of severe undernutrition among under-5 children was around 11.5%, 19.8%, and 12.6% in Bangladesh, Pakistan, and Nepal, respectively. Children from the lowest socioeconomic quintile, and children born with low birth weight were key factors associated with severe undernutrition in these countries. The factors, parental education, maternal nutritional status, antenatal and postnatal care, and birth order were not homogeneous in explaining the determinants of child severe undernutrition across the countries. Our results suggest that the poorest households, and low birth weight of children have significant effects on severe undernutrition among under-5 children in these countries, which should be considered to formulate an evidence-based strategy to reduce severe undernutrition in South Asia.

## Introduction

Child undernutrition refers to deficiencies or imbalances in a child’s intake of energy and/or nutrients^1^. In 2020, approximately 149 million children worldwide aged under five years were estimated to be stunted, with 45 million estimated to be wasted, and 85 million underweight^1^. About 45% of deaths in children are linked to these conditions^1^. Severe undernutrition, especially its acute form is a major cause of death in children under five, and severely undernourished children are often too weak to survive childhood morbidities, such as diarrhoea and pneumonia^2^. Children with severe acute undernutrition are twelve times more likely to die than well-nourished children^3^. However, the overall prevalence of severe undernutrition and its determinants remain to be elucidated.

South Asia is considered vulnerable for its growing number of under-5 severe undernutrition cases, as two-thirds of affected children live in Asia^4^. Among the South Asian counties, Bangladesh, Pakistan, and Nepal are struggling with a high burden of under-5 severe undernutrition (severe stunting) with the prevalence of 11%, 10%, and 10%, respectively between the year 2010 to 2014^5–8^. Despite economic growth and poverty reduction, undernutrition is still rampant in South-Asian countries^9^. This indicates that non-economic factors along with economic development are important. In this study, a nationwide survey from Bangladesh, Pakistan, and Nepal has been used to identify factors that may explain why children under five are severely undernourished.

Under-5 Severe undernutrition is still an issue of discussion. Studies tend to look at disaggregated conventional indicators (e.g., severe stunting, severe wasting, and severe underweight) while it may be better to aggregate these individual indicators^8,10,11^. Conventional disaggregated indicators are not sufficient for quantifying the overall prevalence of severe child undernutrition which was underreported in the previous studies^8,10,11^. These conventional indicators partly overlap, and thus do not provide a convincing estimate of the proportion of undernourished children in the population. Further, prevalence estimates by disaggregating indicators cannot comprehensively capture the burden of undernutrition given that children may suffer from more than one form of undernutrition^12^. The Composite Index of Anthropometric Failure (CIAF) uses conventional undernutrition indicators to provide six different ways of measuring undernutrition^13^. The overall prevalence of undernutrition was estimated by aggregating conventional undernutrition indicators’ values^13^. The CIAF, therefore, convincingly, and inclusively estimates the overall proportion of undernourished children in the population^13^. The Composite Index of Severe Anthropometric Failure (CISAF) in this study has been estimated by using six disaggregated conventional severe undernutrition indicators following the methodological approach of CIAF that provides a convincing estimate of the overall prevalence of severe undernutrition^14^.

Previous studies have identified several maternal, child, contextual and few environmental factors associated with severe undernutrition measured by the CIAF in Bangladesh, India, and other developing countries^15–19^. However, the CISAF was used to measure severe undernutrition in Bangladesh, and certain factors were found to be associated with this condition^20,21^. Further, no previous study used CISAF to explore the prevalence and complex interplay between individual, community, public policy, and environmental risk factors of under-5 severe undernutrition in other South Asian countries including Pakistan and Nepal. Further, available studies used fixed-effect model such as binary logistic regression analysis to identify the determinants of undernutrition or severe undernutrition^20,21^. However, no previous studies addressed the possibility of both community- and household-level effects on severe undernutrition measured by the CISAF in Bangladesh, Pakistan, Nepal as well as other South Asian countries. The present study has considered already known aetiology to identify the associated factors of severe undernutrition as per the CISAF taking into account multilevel random effect (community-, household- and individual- level effects), investigating the change of direction of these factors using more recent data, might help revise important policy decision-making. Therefore, multilevel binary logistic regression with a random intercept was used to identify various factors related to under-5 child severe undernutrition in Bangladesh, Pakistan and Nepal including any community and household variations on severe undernutrition.

## Methods

### Data source and study plan

The Demographic Health Surveys (DHS) are nationally representative household sample surveys that collect population data, health and nutrition, and socioeconomic and anthropometric indicators, emphasising maternal and child health. The DHS is modular in structure, and in addition to the core questionnaire, a set of country-relevant sections and country-specific variables are included. The DHS provides data with standardised variables across surveys. These surveys are administered by ICF International and are nationally representative cross-sectional surveys in low- and middle-income countries^22^.

The DHS used a two-stage stratified sampling technique. Each country was divided into regions, and populations within these regions were stratified by urban and rural areas of residence. Within these stratified areas, enumeration areas were randomly selected based on the most recent population census. In the first stage, primary sampling units were selected from enumeration areas using the probability proportional to size technique, and samples of households were selected in the second stage using the equal probability systematic sampling technique. In the surveys, women of reproductive age (15–49 years) were interviewed to collect children’s information, such as the demography and environment; health and nutrition; and anthropometric data of children born in the five years preceding the interview included. This multistage sampling technique including its sampling weight helps reduce potential sampling bias. Further, the survey interviewers interviewed all ever-married women aged 15–49 years from the pre-selected households without replacement and change in the implementing stage to prevent selection bias^22^. The response rates of interviews in Bangladesh, Pakistan, and Nepal were 99%, 95%, and 96% respectively.

To allow cross-country comparisons, we restricted the samples to the three latest surveys—Bangladesh Demographic and Health Survey (BDHS) 2017–18, Pakistan Demographic and Health Survey (PDHS) 2017–18, and Nepal Demographic and Health Survey (NDHS) 2016, resulting in information on 7902 children under five years of age in Bangladesh, 4227 in Pakistan, and 2379 in Nepal, capturing a period between 2016 and 2018 (Table S1). A detailed list of the survey years by country can be found elsewhere^23–25^.

### Outcome variables

The primary outcome of this study was severe undernutrition among under-5 children, which was measured using the CISAF^14^. A child was considered to be severely stunted (too short stature for age), severely wasted (severely thin for height) and severely underweight (very low weight for age) if the height-for-age, weight-for-height, and weight-for-age indices were three or more Standard Deviations (SDs) below the WHO Child Growth Standards median^26^.

Severe undernutrition indicators for under-5 children were categorized into seven groups: (A) no severe failure; (B) severe wasting only; (C) severe wasting and severe underweight; (D) severe wasting, severe stunting, and severe underweight; (E) severe stunting and severe underweight; (F) severe stunting only; and (Y) severe underweight only (Supplementary Table 2). If children belonged to group (A)—i.e., no severe failure, they were categorized as having no anthropometric failures; otherwise, they were categorized as having any anthropometric failure (i.e., the group from B to Y) (Table S2).

### Independent variables

The potential independent variables were considered based on previous studies^5,6,8,27–29^, and the availability of the dataset. The potential variables were selected from three levels of characteristics: (i) parental characteristics—maternal age (in years) (≤ 24, 25–29, 30–34, ≥ 35), parents’ educational status ((both parents were uneducated (no formal education)), only father was uneducated, only mother was uneducated, both parents were educated), mother’s income-earning status (currently not working, currently working), underweight mother (no, yes), mother received antenatal care (no, yes), mother received postnatal care (no, yes), women’s attitude toward inmate partner violence (not justified, justified), mother’s decision-making autonomy (no, yes); (ii) child characteristics were, for example, children’s age (0–11 months, 12–23 months, 24–35 months, 36–47 months, 48–59 months), sex of child (male, female), birth order (first, second, third, fourth and above), low birth weight (no, yes, not weighted), and child morbidity (no, yes); (iii) household characteristics—source of drinking water (improved, unimproved), solid fuel used in cooking (clean fuel, solid fuel), type of toilet facility (improved, unimproved), mass media exposure (no, yes), wealth index (poorest, poorer, middle, richer, richest), and contextual factors—place of residence (urban, rural) and region of residence (varies for three countries) (see Table S3 for detailed descriptions).

### Statistical analysis

Descriptive statistics were used to present the background characteristics of the children. Since more than 5% missing cases were found in most of the important independent variables (such as, mother received antenatal care, postnatal care, low birth weight etc.) across datasets of three countries, the background characteristics of included and excluded participants were compared and tested if missingness is completely at random (MCAR) using Little’s test, as well as the presence of covariate-dependent missingness^30,31^. We then performed complete case analysis. Covariate-dependent missingness was handled by including those covariates in the models. Bivariate analysis (Chi-square test) was used to explore the associations between the covariates and severe undernutrition among under-5 children. In three countries, variables that were found to be significant at the level *p* < 0.10 in bivariate analysis were included in the multivariable analysis^20,32–34^. In DHS, individuals are nested within households and households are nested within communities, which indicate that individuals, households, and communities are not independent of each other^35,36^. This type of data is usually analysed using a multilevel random intercept model to account for variation in different levels. Therefore, to determine the association between the selected independent variables and the outcome (severe undernutrition as per the CISAF), a multilevel binary logistic regression model with a random intercept term at the community- and household-level were performed. The level of significance for multilevel logistic regression analysis was set at *p* < 0.05, and an odds ratio (OR) with 95% confidence interval (CI) was estimated to determine the associated factors. Stata version 17 (StataCorp LP, College Station, Texas) was used for all analyses considering the complex nature of the sampling weight of all the DHSs. To control the effect of the complex survey design, all bivariate and multivariable analyses of this study were performed using Stata’s “svyset” and melogit commands respectively.

### Model evaluation

The predictive performance (discrimination power) of three multivariable statistical models for three countries respectively, was evaluated by calculating the area under the receiver operation curve (ROC) analysis that determines the accuracy of the models in the prediction of under-5 child severe undernutrition.

### Ethical approval

The data were collected from secondary sources that do not need ethical approval. Informed consent was obtained verbally from each mother of children (every married woman aged 15–49 years) before being enrolled in the study.

## Results

### Background characteristics of the study participants

Complete cases or included participants identified for Bangladesh, Pakistan and Nepal were 4617, 2673, and 1919 respectively (Table S4). The background characteristics of included participants were compared with those who were excluded from this analysis due to missing information on outcomes or covariates. The Little’s test for MCAR based on the significance level (*p* < 0.001) confirmed that the missingness in parents’ educational status, mothers received antenatal and postnatal care, mother’s decision-making autonomy, solid waste and low birth weight (in three countries) were not completely at random. In addition, covariate-dependent missingness was observed; the excluded children were more like to be older age, male, rural settlers and from poor households (Table S5). The findings suggest that the covariates used in the covariate-dependent missingness test could be considered in the final model as well as other important factors might be considered in the complete case analysis.

Furthermore, more than 70% of mothers of children were less than 30 years of age in Bangladesh and Nepal and it was around 53% in Pakistan (Table S4). Parents with no formal education were reported at 50% (22.6 + 27.4) in Pakistan whereas it was around 31.4% and 6% in Nepal and Bangladesh, respectively. More than 45% of children belonged to the socio-economically poorest households in Pakistan and Nepal, and it was 42.3% in Bangladesh. Two-thirds (66.4%) of the children were rural residents in Bangladesh whereas more than half of the children (54%) were in Pakistan and 42.4% in Nepal. Also, more than 50% of the children were less than 2 years of age and were male in the three countries. Detailed information regarding children’s background characteristics is presented in Table S4.

### Prevalence of under-5 severe undernutrition

**Figure 1:**
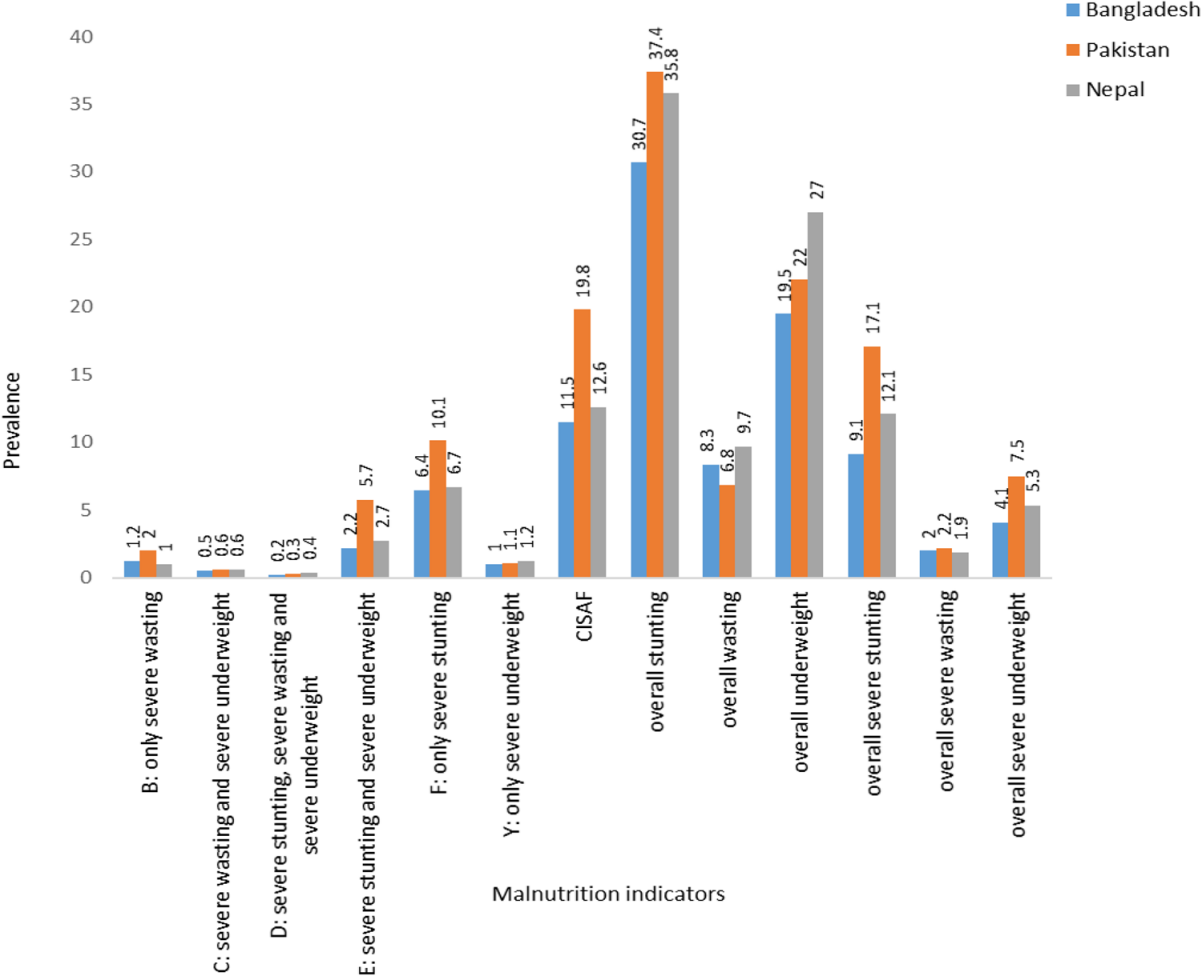
Prevalence of various forms of undernutrition in Bangladesh, Pakistan and Nepal(estimates using complete cases).

Severe stunting, wasting, and being underweight were approximately 9.1%, 2.0% and 4.1% respectively in Bangladesh. Similarly, they are 1.7%, 2% and 7.5% in Pakistan; and 1.2%, 2% and 5% in Nepal respectively (Fig. 1). In the case of a single form, only severe stunting showed a higher percentage in three countries (for example, Bangladesh: 6.4%, Pakistan: 10.1%, and Nepal: 6.7%) whereas the multiple concurrent forms of severe stunting and severe underweight showed higher percentage than other concurrent forms of severe undernutrition (Bangladesh: 2.2%, Pakistan: 5.7% and Nepal: 2.7%) (Fig. 1).

The prevalence of severe child undernutrition based on the CISAF was 11.4%, 19.8%, and 12.6% in Bangladesh, Pakistan, and Nepal, respectively. In Bangladesh, the prevalence was higher among children of parents with no formal education (21.6%), mothers who did not receive antenatal care (18.3%), and children born with low birth weight (18.1%). In Pakistan, a higher prevalence of severe undernutrition was found among children from the poorest households (38%), mothers who did not receive antenatal care (34.4%), and children of parents with no formal education (32%). In Nepal, the prevalence of severe undernutrition was higher among children of the oldest mothers (≥ 35 years) (23.2%), mothers who did not receive antenatal care (25.6%), and children of fourth and above birth order (23.1%) (Table 1).

**Table 1:** Prevalence of under-5 severe child undernutrition in context of socio-demographic factors in Bangladesh, Pakistan, and Nepal.

Characteristics	Bangladesh (n = 4617)		Pakistan (n = 2673)		Nepal (n = 1919)	
	Number	Prevalence (95% CI)	Number	Prevalence (95% CI)	Number	Prevalence (95% CI)
Maternal age (years)						
15–19	288	11.8 (10.0, 13.1)	141	21.8 (17.2, 27.3)	89	9.5 (7.6, 11.8)
20–24	130	10.7 (8.8, 12.9)	165	19.8 (15.6, 24.8)	88	13.5 (10.6, 17.2)
25–29	81	11.6 (9.2, 14.6)	142	17.8 (14.1, 22.3)	45	12.4 (9.0, 17.0)
30–34	39	14.1 (10.0, 19.5)	130	130 (15.2, 24.9)	41	23.2 (15.7, 32.8)
	Χ^2^ = 2.95, *p* = 0.399		χ^2^ = 0.18, *p* = 0.980		χ^2^ = 18.86, *p* = < 0.001	
Parents’ educational status^a^						
Parents with no formal education	31	21.6 (15.3, 29.6)	209	32.0 (26.4, 38.2)	44	20.6 (15.1, 27.4)
Only mother with formal education	90	18.4 (14.8, 22.6)	16	13.3 (6.6, 24.8)	8	9.4 (4.4, 18.9)
Only father with formal education	24	18.1 (11.7, 26.7)	192	22.2 (18.1, 26.8)	71	16.3 (12.5, 21.0)
Both parents with formal education	393	9.9 (8.9, 11.0)	161	13.4 (10.5, 16.8)	140	10.2 (8.4, 12.3)
	χ^2^ = 43.67, *p* = < 0.001		χ^2^ = 123.34, *p* = < 0.001		χ^2^ = 25.65, *p* = < 0.001	
Mother’s working status						
Currently not working	321	10.3 (9.0, 11.7)	509	18.8 (16.2, 21.8)	102	10.7 (8.6, 13.2)
Currently working	217	13.3 (11.6, 15.3)	69	26.1 (19.3, 34.3)	161	14.5 (12.0, 17.3)
	χ^2^ = 2.19, *p* = 0.139		χ^2^ = 0.38, *p* = 0.532		χ^2^ = 5.87, *p* = 0. 0.015	
Underweight mother						
No	419	10.7 (9.6, 12.0)	524	19.4 (16.7, 22.3)	200	10.9 (9.4, 12.7)
Yes	119	15.2 (12.6, 18.3)	54	23.6 ([16.5, 32.7)	63	20.2 (15.4, 26.1)
	χ^2^ = 16.44, *p* = < 0.001		χ^2^ = 1.50, *p* = 0.220		χ^2^ = 10.85, *p* = 0.001	
Mother received antenatal care						
No	66	18.3 (14.0, 23.6)	161	34.4 (28.2, 41.1)	26	25.6 (15.2, 39.9)
Yes	472	10.8 (9.8, 12.0)	417	17.8 (15.2, 20.8)	237	11.9 (10.3, 13.6)
	χ^2^ = 14.81, *p* = < 0.001		χ^2^ = 89.25, *p* = < 0.001		χ^2^ = 9.73, *p* = 0.002	
Mother received postnatal care						
No	133	8.7 (7.1, 10.5)	444	19.5 (16.8, 22.5)	175	12.4 (10.4, 14.7)
Yes	405	12.9 (11.6, 14.4)	134	20.5 (16.3, 25.4)	88	13.0 (10.2, 16.4)
	χ^2^ = 20.10, *p* = < 0.001		χ^2^ = 4.51, *p* = 0.034		χ^2^ = 0.63, *p* = 0.426	
Mother attitude towards wife-beating						
Not justified	422	10.9 (9.7, 12.1)	230	16.9 (13.9, 20.5)	182	12.2 (10.4, 14.2)
Justified	116	14.0 (11.5, 16.8)	348	23.6 (19.9, 27.6)	81	13.7 (10.8, 17.3)
	χ^2^ = 6.03, *p* = 0.014		χ^2^ = 40.84, *p* = < 0.001		χ^2^ = 0.68, *p* = 0.409	
Mother’s decision-making autonomy^b^						
Not practiced	88	11.4 (9.2, 14.1)	309	23.1 (19.4, 27.3)	92	13.2 (10.2, 16.8)
Practiced	450	11.4 (10.2, 12.7)	269	17.2 (14.4, 20.6)	171	12.3 (10.4, 14.6)
	χ^2^ = 0.50, *p* = 0. 476		χ^2^ = 17.87, *p* = < 0.001		χ^2^ = 0.08, *p* = 0.766	
Source of drinking water						
Improved	468	11.8 (10.6, 13.0)	478	18.8 (16.3, 21.6)	225	12.6 (11.0, 14.4)
Unimproved	70	9.4 (7.3, 12.2)	100	26.5 (18.3, 36.8)	38	12.7 (8.6, 18.2)
	χ^2^ = 1.67, *p* = 0.195		χ^2^ = 5.65, *p* = 0.017		χ^2^ = 1.23, *p* = 0.266	
Fuel used in cooking						
Gas and liquid	112	8.2 (6.7, 10.1)	176	14.9 (12.2, 18.2)	47	8.9 (6.3, 12.4)
Solid fuel	426	12.9 (11.5, 14.3)	402	24.5 (20.6, 29.0)	216	14.3 (12.4, 16.4)
	χ^2^ = 25.23, *p* = < 0.001		χ^2^ = 64.01, *p* = < 0.001		χ^2^ = 17.20, *p* = < 0.001	
Type of toilet facility						
Improved	259	10.2 (8.9, 11.6)	405	17.1 (14.6, 19.9)	192	11.5 (9.8, 13.4)
Unimproved	279	13.0 (11.4, 14.8)	173	28.4 (23.2, 34.3)	71	15.5 (12.0, 19.9)
	χ^2^ = 14.38, *p* = < 0.001		χ^2^ = 33.40, *p* = < 0.001		χ^2^ = 2.40, *p* = 0.121	
Mass media exposure						
No	239	14.4 (12.4, 16.7)	292	25.9 (21.3, 31.0)	84	20.4 (16.1, 25.6)
Yes	299	299 (8.7, 11.1)	285	16.4 (13.8, 19.3)	179	10.5 (8.9, 12.3)
	χ^2^ = 18.26, *p* = < 0.001		χ^2^ = 43.88, *p* = < 0.001		χ^2^ = 23.25, *p* = < 0.001	
Wealth index^c^						
Poorest	176	17.2 (14.7, 20.0)	216	37.0 (29.9, 44.8)	114	22.2 (18.1, 26.9)
Poorer	132	13.4 (11.0, 16.3)	173	24.4 (19.3, 30.4)	47	10.8 (7.8, 14.6)
Middle	87	9.7 (7.7, 12.3)	75	12.3 (8.8, 17.0)	47	12.5 (9.0, 17.1)
Richer	83	9.5 (7.5, 12.0)	63	12.7 (8.8, 17.8)	39	9.4 (6.4, 13.6)
Richest	60	6.6 (5.0, 8.6)	51	12.5 (8.7, 17.4)	16	16
	χ^2^ = 67.22, *p* = < 0.001		χ^2^ = 179.73, *p* = < 0.001		χ^2^ = 64.90, *p* = < 0.001	
Place of residence						
Urban	163	10.4 (8.6, 12.6)	209	15.7 (12.5, 19.6)	140	12.1 (10.0, 14.7)
Rural	375	11.8 (10.5, 13.2)	369	21.9 (18.3, 25.9)	123	13.2 (11.0, 15.7)
	χ^2^ = 2.95, *p* = 0.086		χ^2^ = 28.98, *p* = < 0.001		χ^2^ = 2.36, *p* = 0.124	
Age of children (in months)						
0–11	146	7.9 (6.6, 9.6)	119	15.3 (11.6, 19.8)	50	9.7 (7.2, 13.0)
12–23	200	12.3 (10.5, 14.3)	133	15.1 (11.4, 19.8)	72	13.7 (10.4, 17.9)
24–35	192	14.7 (12.6, 17.1)	157	28.9 (23.4, 35.0)	62	14.0 (10.9, 17.7)
36–47			101	24.9 (19.0, 32.0)	40	11.9 (8.6, 16.3)
48–59			68	22.8 (15.9, 31.6)	39	14.7 (9.4, 22.4)
	χ^2^ = 25.54, *p* = < 0.001		χ^2^ = 58.10, *p* = < 0.001		χ^2^ = 7.52, *p* = 0.111	
Sex of child						
Male	303	12.2 (10.8, 13.7)	310	21.3 (17.8, 25.2)	154	13.6 (11.4, 16.3)
Female	235	10.6 (9.1, 12.3)	268	18.2 (15.3, 21.5)	109	11.4 (9.3, 14.0)
	χ^2^ = 1.76, *p* = 0.152		χ^2^ = 1.85, *p* = 0.173		χ^2^ = 2.44, *p* = 0.118	
Birth order						
First	190	10.8 (9.2, 12.7)	105	19.2 (14.6, 24.8)	67	8.6 (6.7, 11.0)
Second	163	10.3 (8.6, 12.2)	110	16.4 (12.3, 21.6)	71	11.0 (8.4, 14.4)
Third	96	11.7 (9.5, 14.2)	93	20.5 (15.4, 26.8])	44	13.7 (9.2, 20.0)
Fourth and above	89	16.1 (12.4, 20.8)	270	21.5 (18.0, 25.5)	81	23.1 (18.1, 29.1)
	χ^2^ = 82.57, *p* = 0.004		χ^2^ 10.03, *p* = 0.018		χ^2^ = 48.01, *p* = < 0.001	
Low birth weight^d^						
No	115	6.4 (5.2, 7.8)	40	11.0 (7.3, 16.3)	107	9.3 (7.5, 11.6)
Yes	57	18.1 (13.8, 23.5)	25	27.3 (16.4, 41.7)	35	16.7 (11.6, 23.4)
Not weighted	366	14.2 (12.6, 15.9)	513	21.0 (18.1, 24.1)	121	16.9 (14.1, 20.2)
	χ^2^ = 82.57, *p* = < 0.001		χ^2^ = 46.45, *p* = < 0.001		χ^2^ = 32.36, *p* < 0.001	
Child morbidity^e^						
No	263	11.4 (9.9, 13.1)	221	19.8 (15.8, 24.5)	180	13.2 (11.2, 15.5)
Yes	275	11.4 (10.0, 13.0)	357	19.8 (16.6, 23.3)	83	11.5 (9.2, 14.3)
	χ^2^ = 0.03, *p* = 0.859	χ^2^ = 1.37, *p* = 0.241	χ^2^ = 2.97, *p* = 0.093			
Total	538	11.4 (10.3, 12.6)	578	19.8 (17.2, 22.6)	263	12.6 (11.1, 14.4)

CI refers confidence interval.

^a^At least five years of schooling (primary level) refers to educated. No (0 years ) schooling year refers to uneducated.

^b^defined as women’s decision making power relative to their male partners.

^c^integrating household asset ownership and access to drinking water and sanitation.

^d^children < 2.5 kg are small.

^e^child had at least one morbid condition out of diarrhea, fever and cough in the two weeks preceding the survey.

#### Regional variations in the prevalence

In Bangladesh, severe undernutrition was highly prevalent among under-5 children in the North-East region (Sylhet division) (13.2%) and the prevalence was lower in the South-West region (Khulna division) (7.8%) (Fig. S1). In Pakistan, the prevalence of severe undernutrition was higher in the South-West region (Baluchistan) (32.4%) and was lower in the North-East region (Federal Capital Territory) (10.2%) (Fig. S2). Again, in Nepal, severe undernutrition was highly prevalent in the mid-Western Development region (Karnail province) (21.5%) and was lower in the Far-Western Development Region (Sudurpashchim province) (10.2%) (Fig S3).

#### Associated factors of under-5 child undernutrition

All multilevel logistic regression models presented in Table 2 were statistically significant (likelihood-ratio χ2 = 113.53, P < 0.001 for Bangladesh; likelihood-ratio χ2 = 285.14, *P* < 0.001 for Pakistan; and likelihood-ratio χ2 = 56.98, *P* < 0.001 for Nepal)^35,36^. The analysis results also showed that the variances of the community and household effects were significant for severe undernutrition measured by CISAF in all three countries with a *p* value of < 0.001 (Table 2).

**Table 2:** Factors associated with under-5 severe child undernutrition in Bangladesh, Pakistan and Nepal (results of stepwise backward regression analysis).

Characteristics	Bangladesh		Pakistan		Nepal	
	AOR (95% CI)	*p* values	AOR (95% CI)	*p* values	AOR (95% CI)	*p* values
Maternal age (years)						
≤ 24	1.00		1.00		1.00	
25–29	0.80 (0.51–1.26)	0.332	0.45 (0.22–0.93)	0.031	1.55 (0.83–2.91)	0.169
30–34	0.78 (0.43–1.41)	0.408	0.31 (0.13–0.75)	0.010	0.95 (0.41–2.22)	0.908
≥ 35	0.76 (0.33–1.73)	0.515	0.15 (0.06–0.43)	0.000	1.88 (0.71–4.98)	0.202
Parents’ educational status						
Parents with no formal education	1.85 (0.84–4.11)	0.128	2.18 (0.94–5.05)	0.069	1.17 (0.53–2.61)	0.697
Only mother with formal education	2.14 (1.33–3.45)	0.002	0.66 (0.18–2.44)	0.532	0.71 (0.18–2.86)	0.629
Only father with formal education	2.02 (0.86–4.71)	0.106	1.75 (0.83–3.67)	0.142	0.81 (0.43–1.53)	0.511
Both parents with formal education	1.00		1.00		1.00	
Mother’s working status						
Currently not working	1.00		1.00		1.00	
Currently working	1.00 (0.71–1.41)	0.988	1.11 (0.51–2.40)	0.796	1.22 (0.74–2.01)	0.440
Underweight mother						
No	1.00		1.00		1.00	
Yes	1.55 (1.02–2.35)	0.038	0.92 (0.39–2.14)	0.844	2.22 (1.25–3.95)	0.006
Mother received antenatal care						
No	1.00		1.00		1.00	
Yes	0.71 (0.42–1.22)	0.220	0.28 (0.13–0.62)	0.001	0.67 (0.27–1.63)	0.374
Mother received postnatal care						
No	1.00		1.00		1.00	
Yes	1.55 (1.06–2.26)	0.024	1.22 (0.69–2.15)	0.501	1.22 (0.75–1.99)	0.423
Mother attitude towards wife–beating						
Not justified	1.00		1.00		1.00	
Justified	1.30 (0.87–1.93)	0.201	1.56 (0.87–2.82)	0.138	0.97 (0.59–1.59)	0.901
Mother’s decision–making autonomy						
Not practiced	1.09 (0.70–1.69)	0.717	1.43 (0.82–2.50)	0.213	1.15 (0.70–1.91)	0.577
Practiced	1.00		1.00		1.00	
Source of drinking water						
Improved	1.00		1.00		1.00	
Unimproved	1.15 (0.60–2.20)	0.665	2.49 (1.07–5.79)	0.034	1.09 (0.48–2.47)	0.836
Fuel used in cooking						
Gas and liquid	1.00		1.00		1.00	
Solid waste	1.59 (0.90–2.81)	0.112	0.45 (0.18–1.11)	0.083	1.22 (0.57–2.62)	0.613
Type of toilet facility						
Improved	1.00		1.00		1.00	
Unimproved	1.10 (0.75–1.61)	0.614	0.75 (0.37–1.52)	0.421	1.42 (0.78–2.57)	0.247
Mass media exposure						
No	1.01 (0.69–1.47)	0.974	0.67 (0.35–1.27)	0.220	1.48 (0.85–2.59)	0.167
Yes	1.00		1.00		1.00	
Wealth index						
Poorest	2.51 (1.14–5.48)	0.022	21.13 (4.84–92.19)	0.000	4.43 (1.38–14.27)	0.013
Poorer	2.09 (1.01–4.31)	0.047	6.95 (1.96–24.60)	0.003	0.98 (0.31–3.06)	0.966
Middle	1.35 (0.68–2.71)	0.390	1.40 (0.46–4.27)	0.551	1.42 (0.48–4.23)	0.525
Richer	1.32 (0.70–2.50)	0.394	0.93 (0.34–2.54)	0.893	1.04 (0.37–2.88)	0.947
Richest	1.00		1.00		1.00	
Place of residence						
Urban	1.00		1.00		1.00	
Rural	0.66 (0.43–1.02)	0.061	0.90 (0.40–2.03)	0.806	0.77 (0.46–1.26)	0.298
Age of children (in months)						
0–11	1.00		1.00		1.00	
12–23	2.15 (1.43–3.24)	< 0.001	0.95 (0.48–1.88)	0.890	1.49 (0.76–2.92)	0.240
24–35	3.16 (2.05–4.86)	< 0.001	8.76 (4.03–19.05)	< 0.001	1.50 (0.74–3.08)	0.263
36–47	–		6.84 (2.90–16.17)	< 0.001	1.21 (0.55–2.65)	0.641
48–59	–		4.07 (1.53–10.80)	0.005	1.25 (0.53–2.91)	0.610
Birth order						
First	1.00		1.00		1.00	
Second	0.99 (0.65–1.51)	0.969	0.67 (0.31–1.44)	0.302	1.39 (0.74–2.63)	0.310
Third	1.01 (0.57–1.78)	0.974	1.29 (0.54–3.11)	0.567	1.24 (0.55–2.79)	0.610
Fourth and above	1.41 (0.71–2.82)	0.330	1.51 (0.64–3.55)	0.342	2.96 (1.14–7.64)	0.025
Low birth weight						
No	1.00		1.00		1.00	
Yes	5.36 (2.80–10.29)	< 0.001	23.34 (5.60–97.23)	< 0.001	2.77 (1.23–6.26)	0.014
Not weighted	2.24 (1.45–3.45)	< 0.001	1.32 (0.55–3.18)	0.540	1.44 (0.83–2.48)	0.193
Child morbidity						
No	1.00		1.00		1.00	
Yes	0.90 (0.66–1.24)	0.533	1.00 (0.60–1.65)	0.969	0.96 (0.59–1.56)	0.875
Level 1 (Household)	0.18 (0.25*)		2.50 (0.76*)		1.24e–35 (6.07e–19*)	
Level 2 (Community)	6.43 (2.34*)		8.65 (1.87*)		5.50 (2.13*)	

aORs with 95% CI were reported from a multilevel logistic regression model accounting for intercept at household_ and community-level.

*Denotes the SE of random intercept and it measures the variability of average effect in each level (community and household) to experience malnutrition. The *P* value for each random effect variance is < .001.

In Bangladesh, the important factors for severe child undernutrition were: children born with low birth weight (OR 5.36, 95% CI 2.80–10.29, *p* < 0.001), versus normal weight; children of age group 24–35 months (OR 3.16, 95% CI 2.05–4.86, *p* < 0.001) versus children of age group 0–11 months; children from the lowest socio-economic quintile (OR 2.51, 95% CI 1.14–5.48, *p* < 0.001) versus the upper socio-economic quintile; and children of parents with no formal education (OR 2.14, 95% CI 1.33–3.45, *p* = 0.002) versus both parents with formal education (Table 2).

Similarly, in Pakistan, children from the lowest socio-economic quintile (OR 21.13, 95% CI 4.84–92.19, *p* < 0.001) versus the upper socio-economic quintile; children born with low birth weight (OR 23.34, 95% CI 5.60–97.23, *p* < 0.001) vs. healthy weight; children less than 3 years of age (24–35 months) (OR 8.76, 95% CI 4.03–19.05, *p* < 0.001) versus children of age group 0–11 months; mothers of oldest age group (20–24 years) (OR 0.15, 95% CI 0.06–0.43, *p* < 0.001) versus the youngest age group (≤ 24 years); children of parents with no formal education (OR 2.49, 95% CI 1.07–5.79) vs. children of parents with formal education were identified key factors associated with severe undernutrition (Table 2).

Further, important factors identified in Nepal were children from the lowest socio-economic quintile (OR 4.43, 95% CI 1.38–14.27, *p* = 0.013) vs. the upper socio-economic quintile; children of fourth and above birth order (OR:2.96, 95% CI 1.14–7.64, *p* < 0.001) vs. first order children; children born with low birth weight (OR 2.77, 95% CI 1.23–6.26, *p* = 0.014) versus healthy weight; and children of underweight mothers (OR 2.22, 95% CI 1.25–3.95, *p* = 0.006) (Table 2).

### Predictive performance

**Figure 2:**
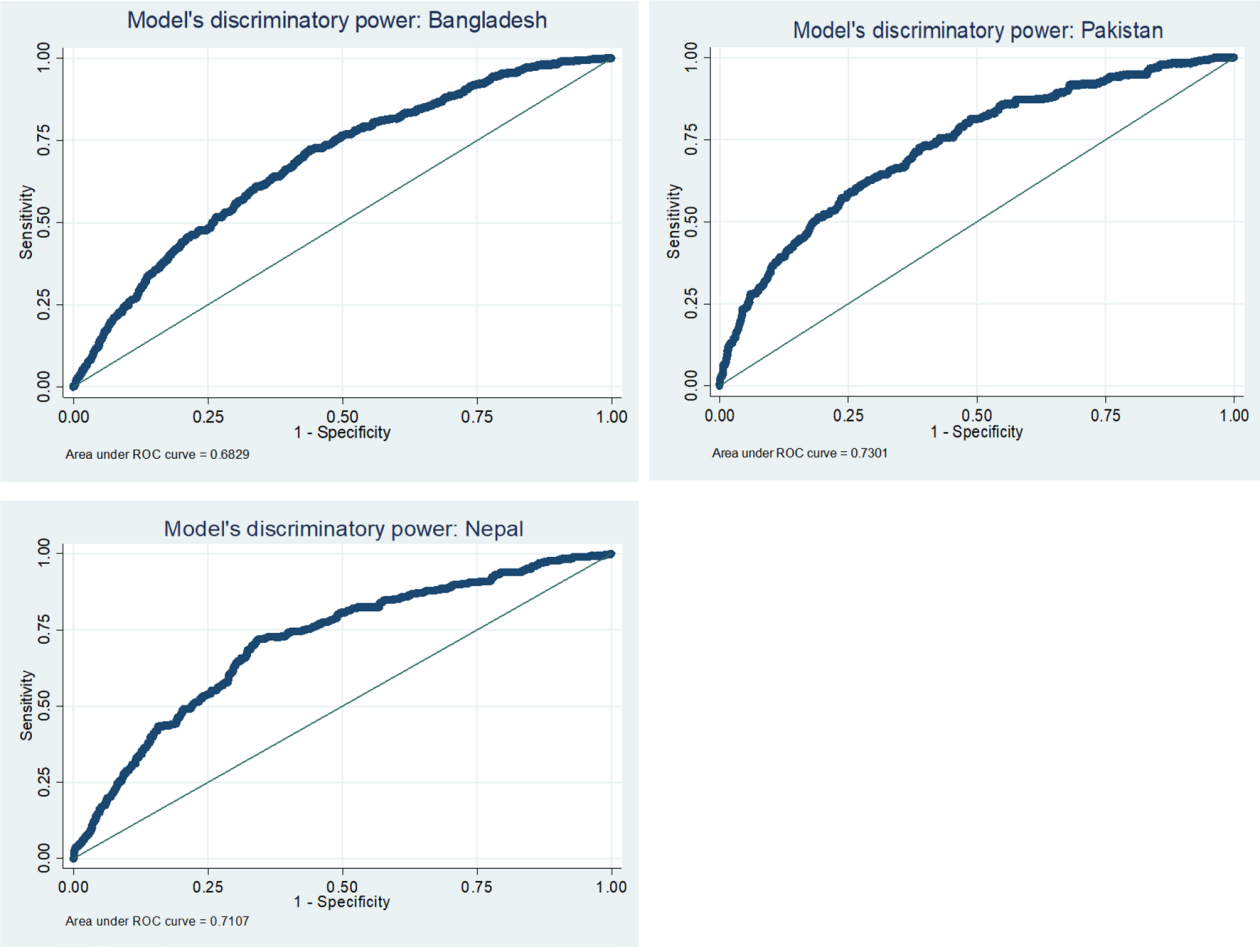
Models’ predictive performance.

Figure 2 described the predictive performance of the three models. The area under the ROC curves for Bangladesh, Pakistan and Nepal were 0.683, 0.730 and 0.711 respectively, indicating the models’ performance in predicting under-5 severe undernutrition was low.

## Discussion

To our knowledge, this is the first study that investigates factors from different levels/perspectives concerning severe undernutrition as per the CISAF taking into account community- and household-level variations for Bangladesh, Pakistan, and Nepal. The study revealed that the overall prevalence of under-5 severe undernutrition was 11.4%, 19.8%, and 12.6% in Bangladesh, Pakistan, and Nepal, respectively. Evidence from 39 low- and middle-income countries showed an average of 20% of severe undernutrition cases were recorded while applying the CISAF method^14^. In the South Asian region, the prevalence of severe undernutrition in India is one of the highest, accounting for more than 16%, based on conventional disaggregated indicators, such as severe stunting, severe wasting, and severe underweight^37^. However, the overall prevalence of severe undernutrition will be higher according to the measurement of the CISAF because it accounts for all forms of anthropometric failure. According to the present findings, the South Asian countries, likes Bangladesh, Pakistan, and Nepal were not very successful in reducing severe child undernutrition. One of the possible reasons could be that there is a lack of coordination between different key sectors. Therefore, coordination between key institutions, for example, government institutions, academic, research and training institutions, and national/international non-governmental organizations is necessary along with addressing other associated factors^38^.

In estimating prevalence, a higher prevalence of severe undernutrition was not shared by the same variable. It differed by children’s parents with no formal education, children from the poorest households, and children of the oldest mothers aged ≥ 35 years in Bangladesh, Pakistan, and Nepal, respectively. On the other hand, the lowest socio-economic quintile, and children born with low birth weight were found to be potential factors associated with severe child undernutrition and common in the three countries, respectively. The findings from the present study are in line with previous studies suggesting that the poorest household have significant effects on under-5 severe undernutrition as per the CISAF in Bangladesh and as per the conventional disaggregated indicators in Pakistan, and Nepal^5,11,20,21,39,40^. Earlier evidence also suggests that the reduction in childhood undernutrition is greater when both the mother and father had higher socioeconomic status^40^. Investing in women’s education, maternal and child healthcare resources and increasing participation of underprivileged people in income-generating activities might be a key to improving the nutritional status of children. Our study also found that children born with low birth weight had a higher chance of severe undernutrition in Bangladesh, Pakistan, and Nepal. Few studies in developing countries assessed low birth weight as a key risk of severe undernutrition using the disaggregated conventional indicator and using CISAF in Bangladesh irrespective of community- and household-level variations^20,21,29,41^. Generally, children who are born with a low birth weight gain inadequate amounts of height and weight^42^. Thus, they may remain shorter and lighter and might suffer from severe undernutrition without adequate nutritional support. More advanced and up-to-date guidelines for antenatal and postnatal care need to be developed and essentially ensured following the guideline by the healthcare provider and mothers that might help to reduce childhood severe undernutrition in those countries.

This study reveals that parental education and children aged 24–35 years of age were found to be associated with severe undernutrition based on the CISAF in Bangladesh and Pakistan whereas mothers’ underweight was associated with severe undernutrition in Bangladesh and Nepal. On top of that, mothers who received postnatal care and children of fourth and above birth order were identified as important correlates of severe undernutrition in Bangladesh and Nepal respectively. The overall burden of severe undernutrition and its distinct factors were not widely articulated while considering community- and household-level variations in Bangladesh, Pakistan, and Nepal. However, the education status of mothers, maternal nutritional status, and birth order have previously been found to be significantly associated with severe acute (wasting) and chronic (stunting) undernutrition in different low and middle-income countries irrespective of community- and household-level variations^43–46^. The present study suggested that various factors associated with severe undernutrition as per the CISAF acted differently across these three South Asian countries and the associated odds ratio (or the magnitude of the risk) varied while considering composite index with community- and household-level variations. Despite sharing similar cultural boundaries, sharp contrasts are appearing in these countries in terms of demography, social context, geography, health, and environment^47,48^. Higher and varied wealth education-based inequalities exist in countries like Bangladesh, Pakistan, and Nepal^47^. In brief, the emerging and varied threats to population structure, society, education, economics, urbanization, environment, geography, and health care might prevent the improvement of the nutritional status of children in the South Asian region, particularly in Bangladesh, Pakistan, and Nepal^47,48^. It is important to consider factors at local, regional, and potentially global levels that help the process of formulating strategies and policies on a transnational basis to reduce severe undernutrition. The present study also suggests introducing community-based management of severe undernutrition for large numbers of children to receive effective nutritional and clinical care in various settings^41^.

A composite index combines multiple indicators of undernutrition (e.g., severe stunting, wasting and underweight) into a single summary measure which provides a more comprehensive and accurate picture of the nutritional status of a population than any single indicator alone. Single indicators can be affected by measurement error. For example, the prevalence of coexistence of stunting and wasting is the interaction term of stunting and wasting^49,50^. Combining multiple indicators into a composite index reduces measurement error and increases the accuracy of undernutrition estimates. While a single indicator may only capture one type of undernutrition, composite index captures different types of undernutrition such as chronic (stunting) and acute (wasting and underweight) undernutrition that can aid in improving the effectiveness of nutritional interventions by targeting multiple forms of undernutrition simultaneously and can have a greater impact on improving children’s health and well-being.

The use of three different national representative household survey data points with a high response rate was a strength of this study. The survey questions and instruments were validated and well-established. A wide range of potential factors from the parental, household, and child perspectives has been used in logistic regression models concerning severe undernutrition among children aged under five years based on the CISAF. In addition, the results from this study are generalizable for Bangladesh, Pakistan, and Nepal, and even for other South Asian countries, because the large sample size accounts for the population across the three countries. However, there are limitations to the study. Due to the cross-sectional nature of the study, it was not possible to establish a causal relationship. Diet practice, ethnicity, and parental behavioural factors were not controlled for in this study due to the lack of availability of this information. Another limitation is that information bias can occur because the survey data was collected by self-reporting information.

## Conclusion

Using the recent Demographic Health Surveys from Bangladesh, Pakistan, and Nepal, our results suggest that children from the poorest households and children born with low birth weight were key factors associated with severe undernutrition. Introducing cost-effective community-based program with a focus on poor and marginalized populations might help reduce under-5 severe undernutrition in South Asian countries. Although the composite index provides a comprehensive scenario, still it is not a popular method in application among demographers and nutritionists. Further extensive research on composite indexing might help to address the high burden of under-5 severe undernutrition comprehensively.

## Supplementary Information

Supplementary Information.

## Data Availability

The data underlying the results presented in the study are publicly accessible and available from the DHS website (https://dhsprogram.com/data/available-datasets.cfm). The name of the datasets are Bangladesh Demographic and Health Survey (BDHS), Pakistan Demographic and Health Survey (PDHS) 2017–18, and Nepal Demographic and Health Survey (NDHS) 2016.

## Abbreviations

BDHS: Bangladesh demographic and health survey
CI: Confidence interval
CIAF: Composite index of anthropometric failure
CISAF: Composite index of severe anthropometric failure
DHS: Demographic health surveys
NDHS: Nepal demographic and health survey
PDHS: Pakistan demographic and health survey
