# Supplementary material for: Prevalence and factors associated with severe undernutrition among under-5 children in Bangladesh, Pakistan, and Nepal: a comparative study using multilevel analysis
Source: Sci Rep. 2023 Jun 22;13:10183. doi: 10.1038/s41598-023-36048-w (PMC10287716; doi:10.1038/s41598-023-36048-w)
Supplement: Supplementary file 1 — Supplementary Information. [file 41598_2023_36048_MOESM1_ESM.docx]

**Supplementary appendix**

**Table S1.** Schematic presentation of sample size selection

| **Indicators** | **Bangladesh**  **(N=7,902)** | **Pakistan**  **(N=4,227)** | **Nepal**  **(N=2,379)** |
| --- | --- | --- | --- |
| Total listed children in DHS surveys | 8,759 | 12,708 | 5,038 |
| Children after excluding dead children | 8,402 | 11,989 | 4,861 |
| Children after excluding absence for height and weight measurement | 7,902 | 4,227 | 2,379 |

**Table S2. Classification of children with severe anthropometric failure**

| **Group name** | **Description** | **Severe Wasting** | **Severe Stunting** | **Severe Underweight** |
| --- | --- | --- | --- | --- |
| A | No severe failure | No | No | No |
| B | Severe wasting only | Yes | No | No |
| C | Severe wasting and severe underweight | Yes | No | Yes |
| D | Severe wasting, severe stunting and severe underweight | Yes | Yes | Yes |
| E | Severe stunting and severe underweight | No | Yes | Yes |
| F | Severe stunting only | No | Yes | No |
| G | Severe underweight only | No | No | Yes |

**Table S3.** Measurements of independent variables

| **Variable** | **Description** | **Measurement** | **Scale of measurement** |
| --- | --- | --- | --- |
| **Mothers’ age (in years)** | Age of mothers at the time of data collection | 15-19, 20-24, 25-29, 30-34, 35-39, ≥ 40 | Discrete, categorial |
| **Parents’ education** | Parental educational status. Accomplishment of at least five years of schooling (primary level, class 1 to 5) refers educated. No (0 years of schooling) schooling year refers uneducated. | Both parents were uneducated, only father was uneducated, only mother was uneducated, both parents were educated | Categorical |
| **Mother’s income-earning status** | Mother engaged in economic activity at the time of data collection | Not working,  Currently working | Binary |
| **Underweight mother** | Mother with <18.5 kg/m^2^ of body mass index refers to underweight, and ≥18.5 kg/m^2^ refers to healthy or not underweight | No, Yes | Binary |
| **Mothers received antenatal care** | At least one medical surveillance and review performed during pregnancy for the early detection of possible complications of pregnancy | No, Yes | Binary |
| **Mothers received postnatal care** | At least one postnatal care is the individualized care provided to meet the needs of a mother and her baby following childbirth | No, Yes | Binary |
| **Mother’s attitudes toward wife-beating** | Respondents are asked if they agree that a husband is justified in hitting or beating his wife under each of the following five circumstances: she burns the food, she argues with him, she goes out without telling him, she neglects the children, and she refuses to have sex with him. If respondents answer “yes” in at least one circumstance, they are considered to have attitudes justifying wife beating. | Not justified, Justified | Binary |
| **Mothers’ decision-making autonomy** | In the BDHS surveys, a woman’s decision-making power is assessed on the following three themes: 1) a woman who usually decides on her healthcare 2) a woman who usually decides on large household purchases and 3) a woman who usually decides on visits to family or relatives. The response options were as follows: (a) respondent alone, (b) respondent and husband/partner, (c) respondent and another person, (d) husband/partner alone, (e) someone else, (f) other. For each question, a value of 1 was assigned for inability in decision-making if the responses were d, e, or f and 0 for otherwise if the responses were a, b, or c. The values were then added, resulting in a score from 0 to 3. The Cronbach’s α for the instruments was 0·79, indicating high internal consistency. | Not participated, Participated | Binary |
| **Source of water** | Improved: piped into dwelling, piped to yard/plot, public tap/standpipe, piped to neighbour, tube well or borehole, protected well, protected spring, rainwater, tanker truck, cart with small tank, bottled water; unimproved: unprotected well, unprotected spring, surface water (river, dam, lake, pond, stream, canal, irrigation channel), other | Improved, unimproved | Binary |
| **Type of toilet facility** | Improved: flush - to piped sewer system, flush - to septic tank, flush - to pit latrine, flush - don't know where, pit latrine - ventilated improved pit, pit latrine - with slab, composting toilet; unimproved: flush - to somewhere else, pit latrine - without slab / open pit, bucket toilet, hanging toilet/latrine, other | Improved, unimproved | Binary |
| **Cooking fuel** | Solid fuel includes coal, lignite, charcoal, wood, straw / shrubs / grass, agricultural crop and animal dung; Clean fuel includes electricity, natural gas, processed gas, biogas, kerosene | Clean fuel, Solid fuel | Binary |
| **Mass media exposure** | Mass media exposure through television, radio and newspaper/magazine has been defined as exposure to at least one media that exposes to at least once a week | No, Yes | Binary |
| **Wealth index** | Wealth index in the DHS surveys is calculated, by the DHS authority, based on information on household characteristics and assets using principal component analysis. Then households are classified into quintiles based on the values of the wealth index, where households with lower values of the index is considered as poorest and vice-versa | Poorest, poorer, middle, richer, richest | Categorical |
| **Children’s age (in months)** | Age of the children at the time of data collection | 0-11 months, 12-23 months, 24-35 months, 36-47 months, 48-59 months | Categorical |
| **Sex of child** | Sex differential of children | Male, Female | Binary |
| **Birth order** | Birth order is the chronological order of sibling births in a family | One, two, three, four and above | Categorical |
| **Low birth weight** | Children were <2.5 kg of weight during birth. Approximately 75% mothers can correctly report their baby's size at birth, therefore mother’s recall is a valid proxy measure of birth weight. | No, Yes, Not weighted | Categorical |
| **Recent morbidity status** | Children had at least cough, fever or diarrhea before 2 weeks of the survey | No, Yes | Binary |

**Table S4.** Compare background characteristics of included and excluded children

| **Characteristics** | **Bangladesh**  **(N=7,902)** | | **Pakistan**  **(N=4,227)** | | **Nepal**  **(N=2,379)** | |
| --- | --- | --- | --- | --- | --- | --- |
|  | **Complete cases**  **(N=4,617)** | **Excluded cases**  **(N=3,358)** | **Complete cases**  **(N=2,673)** | **Excluded cases**  **(N=1,554)** | **Complete cases**  **(N=1,919)** | **Excluded cases**  **(N=460)** |
| **Maternal age (years)** | p <0.001 | | p <0.001 | | p= 0.094 | |
| ≤24 | 2,412 (53.1) | 1,326 (39.5) | 639 (23.9) | 368 (23.7) | 826 (43.1) | 201 (43.7) |
| 25-29 | 1,190 (26.2) | 1,020 (30.4) | 782 (29.3) | 557 (35.8) | 609 (31.7) | 164 (35.6) |
| 30-34 | 675 (14.8) | 651 (19.4) | 656 (24.5) | 394 (25.4) | 303 (15.8) | 66 (14.4) |
| ≥40 | 267 (5.9) | 361 (10.7) | 596 (22.3) | 235 (15.1) | 181 (9.4) | 29 (6.3) |
| **Parents’ educational status** | p =001 | | p=0.026 | | p <0.001 | |
| Uneducated parents | 149 (3.3) | 150 (4.6) | 605 (22.6) | 375 (24.9) | 193 (10.1) | 66 (14.9) |
| Only educated mother | 490 (10.8) | 393 (12.1) | 112 (4.2) | 69 (4.6) | 54 (2.8) | 22 (4.9) |
| Only educated father | 133 (2.9) | 115 (3.5) | 733 (27.4) | 444 (29.6) | 408 (21.3) | 119 (26.9) |
| Educated parents | 3,772 (83.0) | 2,577 (79.7) | 1,223 (45.8) | 614 (40.9) | 1,264 (65.8) | 236 (53.3) |
| **Mother’s current working status** | p <0.001 | | p=0.826 | | p= 0.016 | |
| Not working | 2,843 (62.6) | 1,858 (55.3) | 2,373 (88.8) | 1,374 (88.5) | 877 (45.7) | 239 (52.0) |
| Working | 1,701 (37.4) | 1,500 (44.7) | 300 (11.2) | 178 (11.5) | 1,042 (54.3) | 221 (48.0) |
| **Underweight mother** | p <0.001 | | p=0.260 | | p= 0.016 | |
| No | 3,813 (84.0) | 2,922 (87.0) | 2,456 (91.9) | 1,412 (90.9) | 1,595 (83.1) | 366 (79.6) |
| Yes | 731 (16.0) | 436 (13.0) | 217 (8.1) | 142 (9.1) | 324 (16.9) | 94 (20.4) |
| **Mother received antenatal care** | p=0.370 | | p=0.001 | | p= 0.012 | |
| No | 365 (8.0) | 14 (10.1) | 410 (15.3) | 38 (26.0) | 110 (5.7) | 5 (16.7) |
| Yes | 4,179 (92.0) | 124 (89.9) | 2,263 (84.7) | 108 (74.0) | 1,809 (94.3) | 25 (83.3) |
| **Mother received postnatal care** | p=0.298 | | p=0.322 | | p= 0.104 | |
| No | 1,512 (33.3) | 50 (37.6) | 1,964 (73.5) | 98 (70.0) | 1,235 (64.4) | 15 (50.0) |
| Yes | 3,032 (66.7) | 83 (62.4) | 709 (26.5) | 42 (30.0) | 684 (35.6) | 15 (50.0) |
| **Mothers attitude towards violence** | p =0.365 | | p_=_0.526 | | p= 0.349 | |
| Not justified | 3,737 (82.2) | 2,735 (81.4) | 1,376 (51.5) | 782 (50.4) | 1,369 (71.3) | 318 (69.1) |
| Justified | 807 (17.7) | 623 (18.6) | 1,296 (48.5) | 770 (49.6) | 550 (28.7) | 142 (30.9) |
| **Mother’s decision-making autonomy** | p <0.001 | | p=0.188 | | p <0.001 | |
| Not practiced | 696 (15.3) | 402 (12.4) | 1,223 (45.7) | 782 (50.4) | 687 (35.8) | 205 (46.3) |
| Practiced | 3,848 (84.7) | 2,832 (87.6) | 1,450 (54.3) | 782 (52.0) | 1,232 (64.2) | 238 (53.7) |
| **Source of drinking water** | p <0.001 | | p=0.268 | | p= 0.626 | |
| Improved | 3,868 (85.1) | 2,999 (89.3) | 2,292 (85.8) | 1,351 (86.9) | 1,682 (87.6) | 407 (88.5) |
| Unimproved | 676 (14.9) | 359 (10.7) | 381 (14.2) | 203 (13.1) | 237 (12.4) | 53 (11.5) |
| **Fuel used in cooking** | p=0.004 | | p=0.252 | | p= 0.001 | |
| Gas and liquid | 1,370 (30.1) | 910 (27.2) | 1,204 (45.0) | 670 (43.1) | 549 (28.6) | 97 (29.1) |
| Solid waste | 3,174 (69.8) | 2,442 (72.8) | 1,469 (55.0) | 884 (56.9) | 1,370 (71.4) | 363 (78.9) |
| **Type of toilet facility** | p= 0.034 | | p=0.023 | | p= 0.003 | |
| Improved | 2,534 (55.8) | 1,953 (58.2) | 2,105 (78.8) | 1,176 (75.7) | 1,473 (76.8) | 323 (70.2) |
| Unimproved | 2,010 (44.2) | 1,405 (41.8) | 568 (21.2) | 378 (24.3) | 446 (23.2) | 137 (29.8) |
| **Mass media exposure** | p=0.800 | | p=0.268 | | p= 0.001 | |
| No | 1,641 (36.1) | 1,222 (36.4) | 1,036 (38.7) | 626 (40.4) | 398 (20.7) | 128 (27.8) |
| Yes | 2,903 (63.9) | 2,136 (63.6) | 1,637 (61.3) | 40.4 (59.6) | 1,521 (79.3) | 332 (72.2) |
| **Wealth index** | p=0.604 | | p=0.016 | | p= 0.011 | |
| Poorest | 991 (21.8) | 776 (23.1) | 550 (20.6) | 355 (22.8) | 464 (24.2) | 133 (28.9) |
| Poorer | 933 (20.5) | 664 (19.8) | 656 (24.5) | 395 (25.4) | 420 (21.9) | 94 (20.5) |
| Middle | 828 (18.2) | 602 (17.9) | 504 (18.9) | 302 (19.4) | 401 (20.9) | 110 (23.9) |
| Richer | 913 (20.1) | 652 (19.4) | 469 (17.6) | 277 (17.8) | 391 (20.4) | 87 (18.9) |
| Richest | 897 (19.4) | 664 (19.8) | 494 (18.5) | 225 (14.5) | 243 (12.6) | 36 (7.8) |
| **Place of residence** | p=0.266 | | p=0.497 | | p= 0.004 | |
| Urban | 1,526 (33.6) | 1,168 (34.8) | 1,229 (46.0) | 696 (44.8) | 1,105 (57.5) | 231 (50.2) |
| Rural | 3,018 (66.4) | 2,190 (65.2) | 1,444 (54.0) | 858 (55.2) | 814 (42.4) | 229 (49.8) |
| **Age of children** | p<0.001 | | p<0.001 | | p<0.001 | |
| 0-11 months | 1,665 (36.6) | 64 (1.9) | 800 (29.9) | 58 (3.7) | 466 (24.3) | 7 (1.5) |
| 12-23 months | 1,544 (34.0) | 80 (2.4) | 718 (26.9) | 110 (7.1) | 494 (25.7) | 17 (3.7) |
| 24-35 months | 1,333 (29.2) | 193 (5.7) | 522 (19.5) | 324 (20.9) | 379 (19.8) | 84 (18.3) |
| 36-47 months | 2 (0.04) | 1,461 (43.5) | 368 (13.8) | 487 (31.3) | 330 (17.2) | 151 (32.8) |
| 48-59 months |  | 1,560 (46.5) | 265 (9.9) | 575 (37.0) | 250 (13.0) | 201 (43.7) |
| **Sex of children** | p= 0.684 | | p= 0.445 | | p<0.001 | |
| Male | 2,381 (37.8) | 1,744 (51.9) | 1,369 (51.2) | 777 (50.0) | 1,038 (54.1) | 207 (45.0) |
| Female | 2,163 (47.6) | 1,614 (48.1) | 1,304 (48.8) | 777 (50.0) | 881 (45.9) | 253 (55.0) |
| **Birth order** | p= 0.294 | | p<0.001 | | p<0.001 | |
| First | 1,720 (37.9) | 1,299 (38.7) | 535 (20.0) | 512 (32.9) | 713 (37.2) | 223 (48.5) |
| Second | 1,502 (33.0) | 1,071 (31.9) | 582 (21.8) | 312 (20.1) | 585 (30.5) | 109 (23.7) |
| Third | 778 (17.1) | 549 (16.3) | 457 (17.1) | 260 (16.7) | 298 (15.5) | 65 (14.1) |
| Fourth and above | 544 (12.0) | 439 (13.1) | 1,099 (41.1) | 470 (30.2) | 323 (16.8) | 63 (13.7) |
| **Low birth weight** | p<0.001 | | p<0.001 | | p<0.001 | |
| No | 1,765 (38.8) | 94 (27.7) | 432 (16.2) | 187 (12.0) | 1,086 (56.6) | 205 (44.6) |
| Yes | 303 (6.7) | 35 (10.3) | 99 (3.7) | 38 (2.4) | 166 (8.6) | 31 (6.7) |
| Not weighted | 2,476 (54.5) | 210 (62.0) | 2,142 (80.1) | 1,327 (85.5) | 667 (34.8) | 224 (48.7) |
| **Child morbidity** | p<0.001 | | p<0.001 | | p<0.001 | |
| No | 2,205 (48.5) | 1,960 (48.4) | 1,076 (40.2) | 1,076 (40.2) | 1,262 (65.8) | 364 (79.1) |
| Yes | 2,339 (51.7) | 1,395 (41.6) | 1,597 (59.8) | 1,597 (59.8) | 657 (34.2) | 96 (20.9) |
| Little’s test for MCAR | p<0.001 | | p=0.0002 | | p<0.001 | |

Note: MCAR denotes Missing Completely At Random,

p values were generated from chi-square test

**Table S5** - Little’s test for Missing Completely at random and test for covariate-dependent missingness

| **Models**^†^ | **Covariates** | **Bangladesh** | **Pakistan** | **Nepal** |
| --- | --- | --- | --- | --- |
|  |  | **p-values** | **p-values** | **p-values** |
| Model 1 | No covariates | <0.001 | 0.0002 | <0.001 |
| Model 2 | Age of child | 0.999 | 0.062 | 0.040 |
| Model 3 | Age of child and place of residence | 0.998 | 0.063 | 0.112 |
| Model 4 | Age of child, place of residence and wealth index | 0.999 | 0.930 | 0.461 |
| Model 5 | Age of child, sex of child, place of residence and wealth index | 0.999 | 0.918 | 0.560 |

Note: ^†^Models 2-5 were used to test the assumption

**Figures**


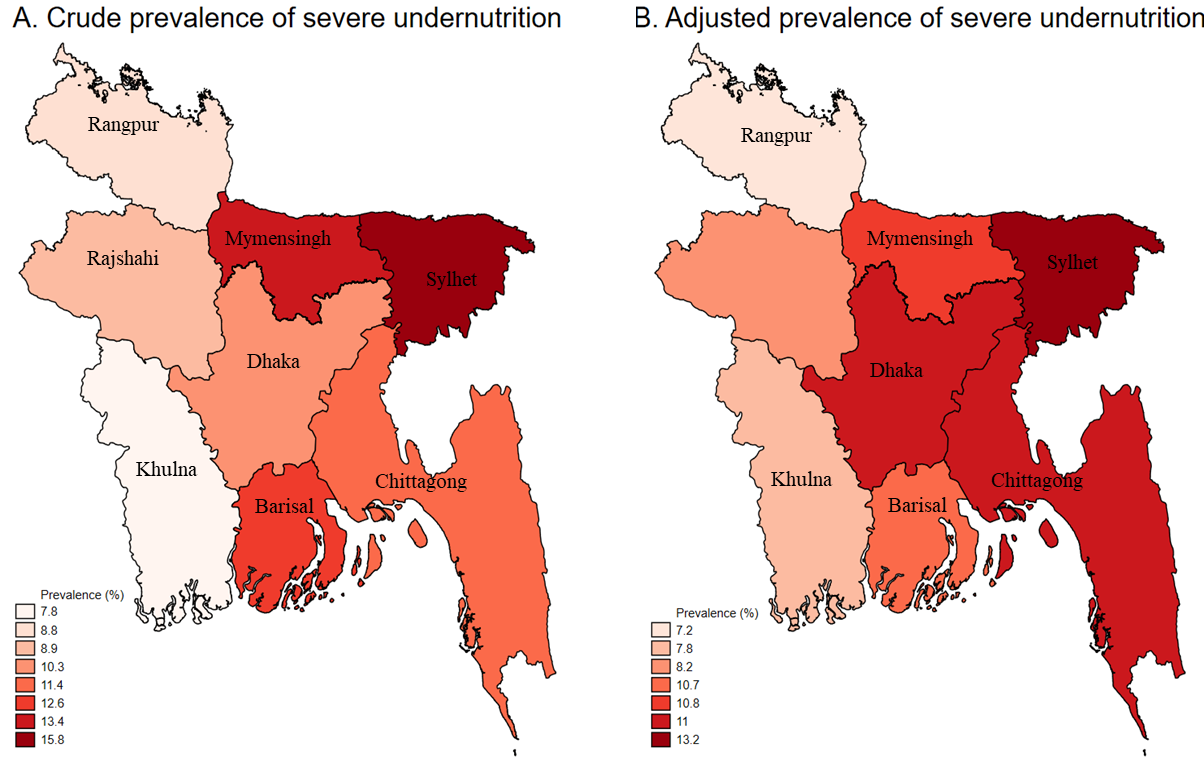


**Supplementary** **Figure S1**: Overall prevalence of severe undernutrition in Bangladesh.

*Note:* Prevalence was adjusted for the variables found significant in the regression model


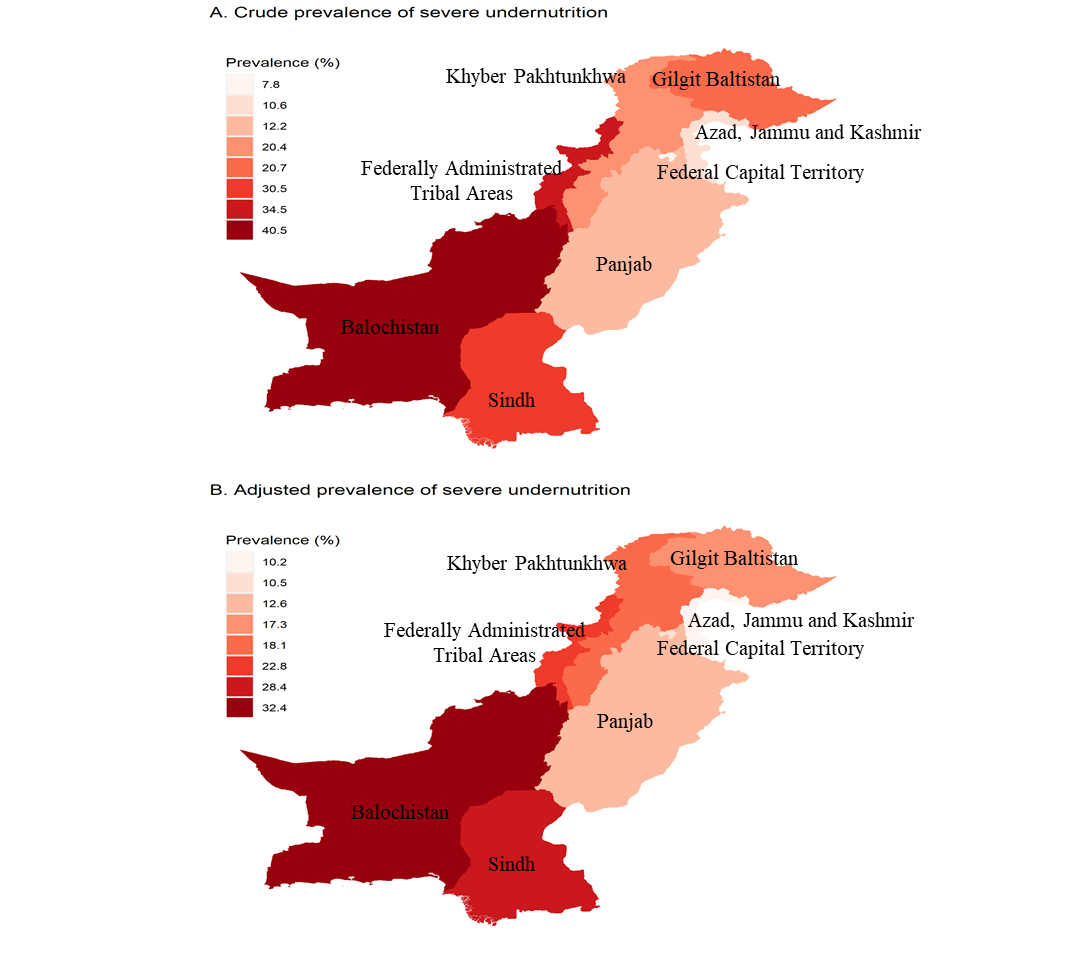


**Supplementary** **Figure S2**: Overall prevalence of severe undernutrition in Pakistan.

*Note:* Prevalence was adjusted for the variables found significant in the regression model


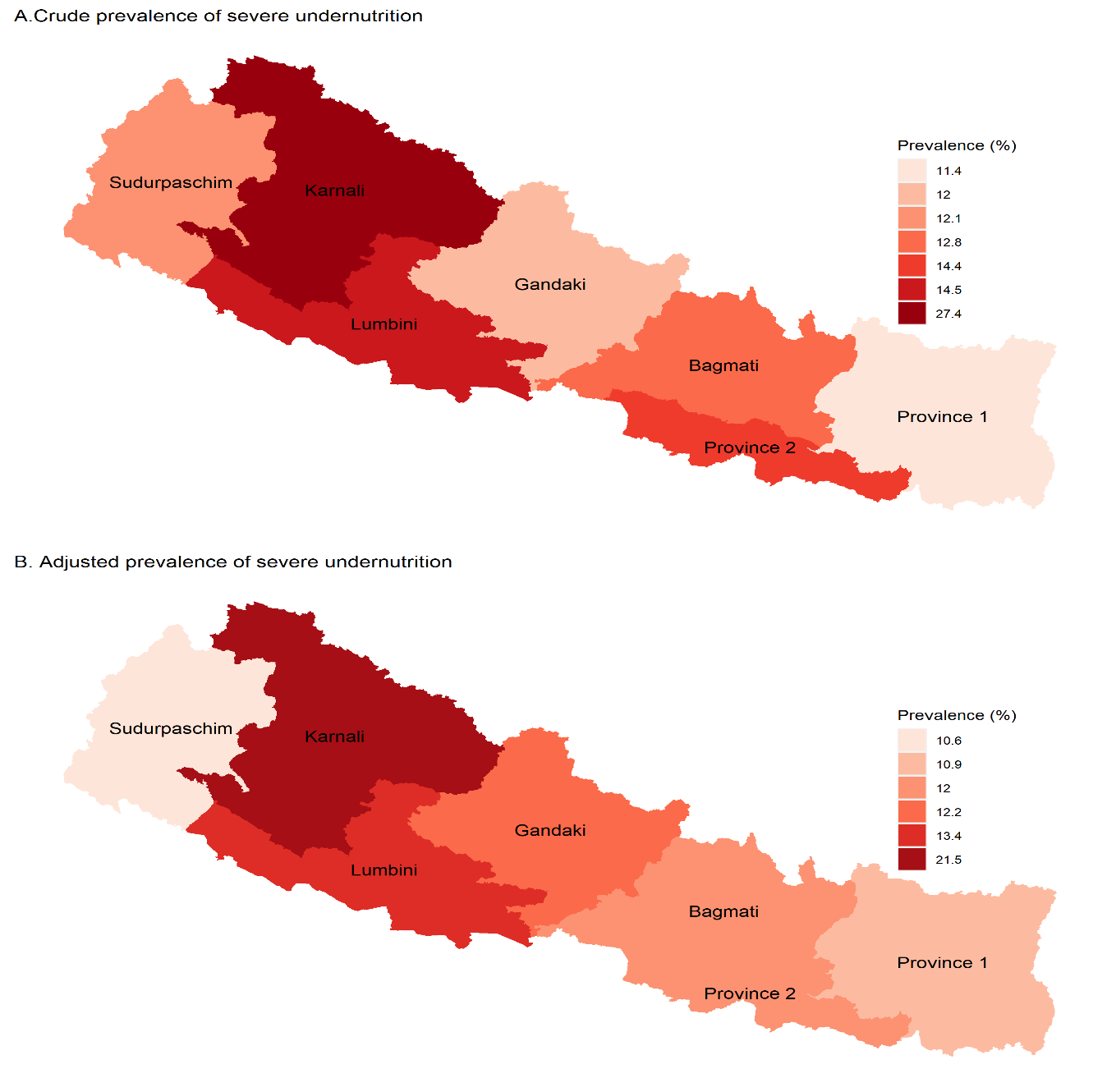


**Supplementary** **Figure S3**: Overall prevalence of severe undernutrition in Nepal.

*Note:* Prevalence was adjusted for the variables found significant in the regression model
